# By the Way

**Published:** 1910-08-13

**Authors:** 


### By the Way.

### THE MALADY OF FRANCIS I.

Antoine Le Coq, a physician of Paris, having been called into consultation with reference to the condition of Francis I., who was suffering from venereal disease, strongly disagreed with the advice of Fernel, who wished to treat the patient with his own "anti-venereal opiate." Le Coq insisted on the use of mercurial inunctions as the most speedy and most certain remedy. "He is a rascal," said he, referring to the King, "he is a rascal who has caught the pox. *Frottetur*, let him be rubbed like any other, and like the humblest of his subjects, since he has disfigured himself in the same manner." This saying was repeated to Francis, who, it is reported, only laughed at it, and took it in good part.—Dr. Witkowski in *Anecdotes Médicales*.

### THE SALUTARY EFFECTS OF FEAR.

In his *Mélanges de Chirurgie*, the celebrated lithotomist of Lorraine, Nicolas Saucerotte, who in 1794 was chief surgeon to the Army of Sambre-et-Meuse, relates in the following anecdote how a great fright may sometimes have good effects. "A woman suffering for eighteen months from prolapse of the uterus had been treated unsuccessfully by all known methods. She was about to be given up as incurable, when as a last resource a simple stratagem was tried. A mouse having been caught, a cord was attached to its foot, and it was made to run about in the patient's skirts without her knowing anything of what was going to be done. She was so startled at feeling the animal climbing up her legs that she jumped up suddenly and ran out of the room like one possessed. As the result of the emotion produced by this unexpected stimulus applied suddenly the womb returned to its normal position, and the woman was cured." Moreover, is it not well known that a great fright gives wings to laggards, speech to the dumb, and the use of their limbs to paralytics? Herodotus tells the story of the son of Crœsus, who, being dumb, recovered his voice the moment he saw a Persian with a naked dagger throw himself upon his father. "Stop, soldier; do not kill Crœsus," cried he; and from that time he spoke! Valleriola also cites the story of an old paralytic who, on being surrounded by flames, made a supreme effort to rise up and flee from his burning room. In this way he recovered his power of locomotion, "which he had the pleasure of enjoying for the rest of his life." And the worthy Saucerotte adds with frankness: "It is the duty of an intelligent surgeon to appreciate the circumstances in which it is proper for him to put this affection of the soul into play without danger."—*Dr. Bonnente in "La Chronique Médicale."*

